# Genetic and epigenetic alterations at secondary resistance after continued decitabine-based treatment of acute myeloid leukemia in the randomized phase II DECIDER trial

**DOI:** 10.1038/s41375-025-02780-7

**Published:** 2025-10-07

**Authors:** Inga Hund, Maria Elena Hess, Geoffroy Andrieux, Julia Stomper, Gabriele Greve, Christoph Niemöller, Tobias Ma, David Uhl, Olga Grishina, Felicitas Thol, Michael Heuser, Gesine Bug, Martina Crysandt, Andreas Neubauer, Justus Duyster, Hartmut Döhner, Melanie Boerries, Michael Lübbert, Heiko Becker

**Affiliations:** 1 Department of Hematology, Oncology and Stem Cell Transplantation, Medical Center - University of Freiburg, Faculty of Medicine, University of Freiburg, Freiburg, Germany; 2 Faculty of Biology, University of Freiburg, Freiburg, Germany; 3 Institute of Medical Bioinformatics and Systems Medicine, Medical Center - University of Freiburg, Faculty of Medicine, University of Freiburg, Freiburg, Germany; 4 Department of Hematology, Oncology, Hemostaseology and Stem Cell Transplantation, University Hospital RWTH Aachen, Aachen, Germany; 5 Institute of Genetic Epidemiology, Faculty of Medicine and Medical Center - University of Freiburg, Freiburg, Germany; 6 Clinical Trials Unit, Medical Center-University of Freiburg, Freiburg, Germany; 7 Department of Hematology, Hemostasis, Oncology and Stem Cell Transplantation, Hannover Medical School, Hannover, Germany; 8 Department of Internal Medicine IV, University Hospital Halle (Saale), Martin-Luther-University Halle-Wittenberg, Halle, Germany; 9 Department of Medicine II, Hematology and Oncology, University Hospital Frankfurt, Goethe University, Frankfurt, Germany; 10 Department of Hematology and Oncology, University Clinic Gießen/Marburg, Marburg, Germany; 11 German Cancer Consortium (DKTK), Partner site Freiburg, a partnership between DKFZ and Medical Center - University of Freiburg, Freiburg, Germany; 12 Department of Internal Medicine III, University Hospital of Ulm, Ulm, Germany

**Keywords:** Translational research, Acute myeloid leukaemia, Cancer epigenetics

Therapies containing the hypomethylating agents (HMA) decitabine (DEC) or azacitidine (AZA) are standard of care for patients with acute myeloid leukemia (AML) ineligible for intensive chemotherapy. However, initially responsive disease eventually develops secondary resistance [1–4].

Resistance against DEC may result from alterations in pyrimidine metabolism, e.g., deficiency of the DEC-activating deoxycytidine kinase (DCK), or upregulation of the DEC-catabolizing cytidine deaminase or upregulation of SAMHD1, inactivating DEC triphosphate [5–8]. Moreover, expansion of subclones with mutations in signaling proteins may cause secondary resistance [9]. However, for most patients, the resistance-mediating alteration remains elusive.

Here, we investigated acquired resistance after continued DEC treatment in the randomized phase II DECIDER trial.

In this trial, 200 patients received DEC alone or combined with valproic acid (VPA) or all-*trans* retinoic acid (ATRA) or VPA and ATRA [4]. Thirty-five (18%) patients achieved a complete remission (CR), CR without recovery of platelets or neutrophils (CRi), or partial remission (PR); further, 84 (42%) patients had an anti-leukemic effect (ALE) or stable disease (SD). Of these, 14 patients with progressive disease (PD) or relapse after ≥6 months of treatment had samples available from both treatment start and time of PD. Pretreatment characteristics are provided in Supplementary Table S1. Patient selection and methods are described in the Supplementary Data.

All 14 patients received DEC, 8 also received ATRA, and 4 VPA. Six patients achieved CR, CRi or PR as best response, the remainder ALE or SD (Fig. 1). Resistance samples were collected a median of 10.1 months after treatment initiation (range: 6–64), corresponding to a median of 9 treatment cycles (range: 7–52), including 7 patients with ≥12 cycles (Fig. 1).

**Fig. 1:**
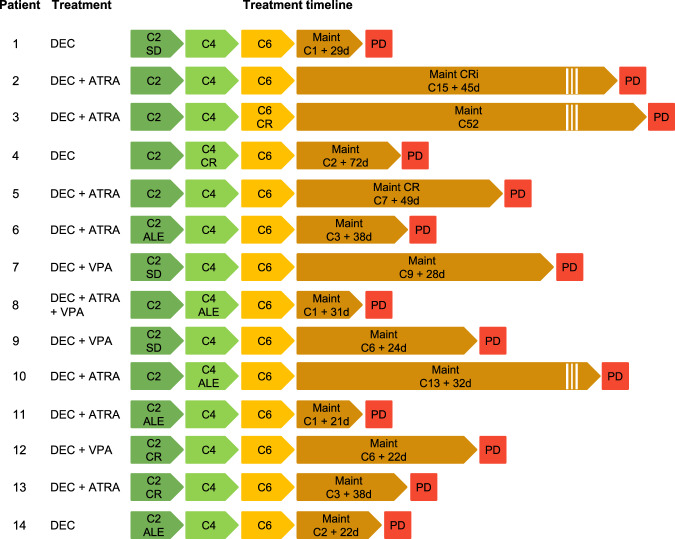
Treatment and treatment timeline of patients. Depiction of the treatment each patient received and the time on treatment until sampling. Best response indicated per patient. ALE anti-leukemic effect, ATRA all-*trans* retinoic acid, C cycle, CR complete remission, CRi CR without recovery of platelets or neutrophils, DEC decitabine, Maint maintenance, PD progressive disease, PR partial remission, SD stable disease, VPA valproic acid.

We analyzed blasts collected at baseline and at resistance via whole exome sequencing (WES). At baseline, patients harbored a median of 16 mutations (range: 3–25). Most frequently mutated was *RUNX1* (*n* = 4 patients); two patients each harbored mutations in *ASXL1*, *SF3B1*, *SRSF2*, or *TET2*.

At secondary resistance, the overall number of mutations had increased to a median of 36 (range: 6–63), indicating a clonal shift towards resistance. We concentrated on genes known to be cancer-associated (as defined by OncoKB) and on recurrently mutated genes (i.e., acquired in ≥2 patients) (Fig. 2A).

**Fig. 2:**
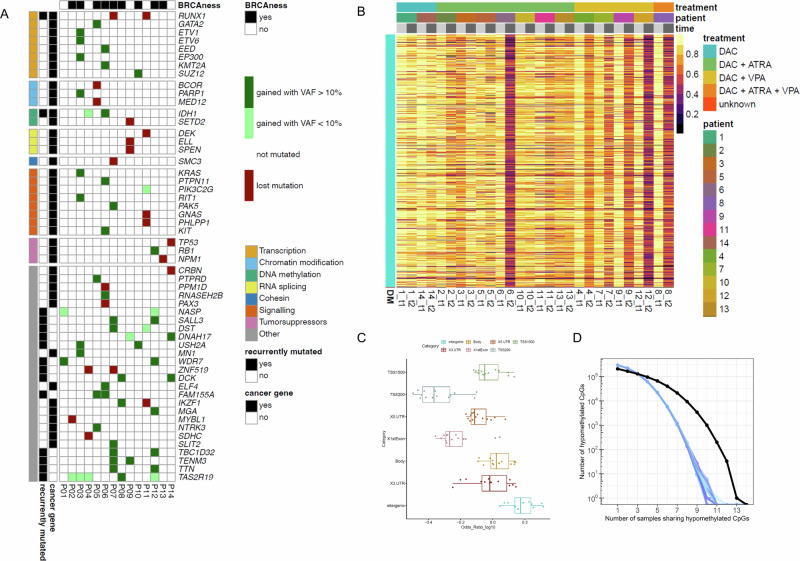
Changes in mutation and DNA methylation at secondary resistance. **A** Overview of mutated cancer genes (according to OncoKB) and recurrently mutated genes gained or lost at secondary resistance compared with baseline for each patient. Acquisition of AC03 signature (BRCAness) is indicated in the upper row. VAF, variant allele frequency. **B** Comparative heatmap displaying methylated CpG sites for each patient at treatment start (t1) and resistance (t2). Group-wise differential analysis to show methylation changes between baseline (t1) and resistance (t2). The x-axis represents individual patients, and the y-axis corresponds to CpG sites showing hypomethylation. Patients are grouped by treatment (as indicated). **C** Genomic distribution of differentially methylated CpGs. Hypomethylated sites were annotated based on their genomic context and assessed for their enrichment relative to the overall distribution of these regions in the EPIC array. Enrichment of hypomethylated CpGs of the intergenic region can be seen, as well as depletion of hypomethylation in the TSS200 promoter regions. **D** Identification of shared hypomethylated CpGs across patients. Significant non-random overlap of hypomethylated CpGs (*p* value 7.03E-09). Blue lines are random CpGs from the EPIC array, whereas the black lines are CpGs commonly hypomethylated across patients. Of overall 208, 248 hypomethylated CpGs, 21,235 were consistently hypomethylated in >50% of patients. ATRA all-*trans* retinoic acid, DEC decitabine, VPA valproic acid.

Towards resistance, nine patients had gained at least one mutation in a cancer gene, corresponding to a median of one mutation per patient (range: 0–7). *IDH1* was the only cancer gene that acquired a mutation in more than one patient (p.R132G VAF 5.3%, p.R132H 48%). Further cancer genes mutated in single samples are involved in signaling pathways (e.g., *KIT* VAF 21%*, KRAS* 30%), or transcription (e.g., *GATA2* VAF 45%*, KMT2A* 43%).

Mutations in recurrently mutated genes were acquired in 13 patients, with a median of 2 per patient (range: 0–6). In addition to *IDH1* mutations, mutations in *DCK* (p.S13X VAF 17%, p.R104G VAF 11%) were acquired at resistance in two patients. Further recurrently mutated genes included *SALL3* (VAFs 29%, 22%)*, USH2A* (VAFs 36%, 39%), and *TAS2R19*.

*TAS2R19* mutations were acquired in five patients at two distinct loci (p.K167E VAF 6.3%, 11%; p.G77S 7.2%, 6.1%, 6.3%). *TAS2R19* has no known function in AML; it is predicted to be involved in G protein-coupled receptor signaling pathways and is rarely mutated in cancer (Supplementary Table S2). Recurrence and hotspot location, but not the low VAF, support the functional relevance of these mutations.

Thus, in individual patients, resistance may be linked with mutations in genes with well-established roles in AML or HMA metabolism [5–9]. To our knowledge, ours is the first report to identify mutant *DCK* as potential secondary resistance mechanism in DEC-treated patients. Future studies may investigate whether switch to AZA (activated through uridine-cytidine kinases, not DCK) may be beneficial in these patients. In the absence of 2nd line options, mutations in signaling pathways may support the switch to respective inhibitors. Mutant *IDH1* may offer the ready possibility for salvage treatment with an IDH1 inhibitor.

At secondary resistance, we also observed the loss of a median of six mutations (range: 1–15) per patient compared to baseline. These included mutations in *RUNX1* (baseline VAFs 35%, 99%), *NPM1* (26%)*, PPM1D* (12%), and *TP53* (95%) (Fig. 2A).

Given the latter observation and the attention *TP53* receives in the context of DEC [10], we studied *TP53*, including copy number analyses, in greater detail (Supplementary Fig. S2). The *TP53* mutation loss occurred in patient 14 (P14), who was the only patient with mutant *TP53* at baseline and who had a single *TP53* copy at baseline (mutant) and resistance (wild-type). Two other patients (P8, P12) gained *TP53* wild-type alleles towards resistance. Two patients (P9, P10) exhibited bi-allelic *TP53* losses at both baseline and resistance. That patients would rather acquire *TP53* wild-type alleles than *TP53* alterations under long-term DEC treatment is unlikely related to resistance but highlights the unique and incompletely understood impact of DEC on *TP53*-mutant AML clones.

To better comprehend the data, we determined signatures of cancer processes from the WES data (Supplementary Fig. S3) [11]. Among these, signature AC03 was gained towards resistance in nine patients (Fig. 2A, Supplementary Fig. S3). The acquisition correlated with treatment duration and VPA co-treatment, although not statistically significant (Supplementary Fig. S4). The AC03 gain was also identified in all cell lines with secondary DEC resistance we generated (Supplementary Fig. S5).

AC03 resembles the mutational phenotype of *BRCA1*/*BRCA2*-mutant cancers, despite lacking these mutations (i.e., BRCAness), due to alternative mechanisms of homologous recombination deficiency [12]. Cancers with BRCAness rely on PARP1 and can be targeted by PARP inhibitors (PARPi). DEC enhances PARP1 chromatin recruitment, synergizing cytotoxicity with PARPi [13]. A phase I trial of DEC plus the PARPi talazoparib (TAL) in AML (22 of 25 patients had prior HMA) showed increased PARP trapping and γH2AX foci in responders [14].

Given the BRCAness at DEC resistance and clinical availability of PARPi, we tested whether cell lines with acquired BRCAness show increased sensitivity to TAL (Supplementary Fig. S6). TAL alone did not induce apoptosis in any tested cell lines, including those with secondary DEC resistance, nor did it increase apoptosis in combination treatments, compared to controls. Solely, in treatment-naive MOLM13 cells, apoptosis increased with TAL + DEC and TAL + DEC + ATRA (Supplementary Fig. S7). Differentiation marker analyses also revealed no major impact of TAL alone or combined (Supplementary Fig. S6).

The lack of increased PARPi sensitivity suggests that BRCAness after DEC treatment may not stem from HRd. Consistently, BRCAness was not associated with high (>42) HRd scores in patient samples (Supplementary Table S3). While corroboration outside of cell lines (e.g., patient-derived xenograft models) is needed, our results suggest that HMA-induced genomic instability leads to BRCAness (defined by AC03 signature) that may not predict PARPi sensitivity.

We assessed DNA methylation profiles and compared them at resistance to baseline using single-sample analysis (Fig. 2B). A median of 29,264 CpGs (8.9%) were hypomethylated at resistance, whereas hypermethylation was less frequent (median 9853 CpGs, 3%). Consistent with single-sample results, group-wise analysis found 17,854 CpGs (5.4%) significantly hypomethylated at resistance, with only two CpGs significantly hypermethylated. This indicates the lasting biologic effect of long-term DEC treatment, persisting even at secondary resistance.

Further analyses revealed no significant correlations between the number of hypo- and hypermethylated CpGs and treatment duration (Supplementary Fig. S8). Analyses of the genomic distribution of differentially methylated CpGs showed a small but significant enrichment in intergenic regions and depletion near the promoter regions (TSS200) (Fig. 2C, Supplementary Fig. S9). We next investigated shared hypo- and hypermethylated CpGs across patients and noted a non-random overlap among hypomethylated CpGs, but not hypermethylated CpGs (Fig. 2D, Supplementary Fig. S9).

Gene set enrichment analysis showed the top 10 most enriched terms for hypomethylated CpGs were related to ion transport or the nervous system (Supplementary Fig. S10). Overactive ion transport in AML is understudied. Among the hypomethylated genes at resistance was *SLC39A10* (zinc transporter ZIP10); blocking ZIP10 decreases AML cell growth and viability [15].

In the DECIDER trial, we observed that adding ATRA delays secondary resistance [4], leading to overrepresentation of respective patients in our current study. The beneficial impact of ATRA combined with DEC is being further investigated in the DECIDER-2 trial (DRKS00023646). Due to relatively low patient numbers, we could not analyze mutational or methylation differences by DEC combination partners, though ATRA and VPA may impact the molecular underpinnings of resistance development in the individual patient. Despite this, it may even be conceivable that our data can be applied to the current HMA + venetoclax standard, but future studies are required to confirm this.

Our study is the first to provide extensive genetic and DNA methylation data on AML patients with secondary resistance after prolonged HMA treatment. While no universal gene or pathway was linked to resistance, individual patients acquired mutations with biological or clinical relevance (e.g., in *DCK*, *IDH1,* or signaling genes). In addition, we observed BRCAness emerging in most patients, likely rather as product of continued DEC treatment than driving resistance, and not sensitizing to PARPi in cell lines. DNA methylation profiling identified CpGs differentially methylated between baseline and resistance, comprising almost exclusively hypomethylated CpGs at resistance. Persistent and non-random DNA hypomethylation at resistance may inform future treatment approaches.

## Supplementary information

Supplementary Data/Supplemental Material

## Data Availability

Due to ethical and legal considerations, patient data from this study cannot be shared publicly. Qualified researchers may request access to anonymized data by contacting heiko.becker@uniklinik-freiburg.de and providing a detailed data access proposal, subject to approval by the relevant ethics committee.
