# Supplementary material for: Genetic and epigenetic alterations at secondary resistance after continued decitabine-based treatment of acute myeloid leukemia in the randomized phase II DECIDER trial
Source: Leukemia. 2025 Oct 7;39(11):2816–9. doi: 10.1038/s41375-025-02780-7 (PMC12589117; doi:10.1038/s41375-025-02780-7)
Supplement: Supplementary file 1 — Supplementary Data/Supplemental Material [file 41375_2025_2780_MOESM1_ESM.pdf]

Supplementary Data

Genetic and epigenetic alterations at secondary resistance after continued decitabine-based treatment of acute myeloid leukemia in the randomized phase II DECIDER trial

Hund et al.

Supplementary Methods

Patient samples

To be included in the DECIDER trial (NCT00867672), patients had to be over 60 years of age, have a newly diagnosed AML (non-M3), be ineligible for induction chemotherapy and have an ECOG performance status (PS) of 0-2. Treatment (2x2 factorial design) was as follows: DEC 20 mg/m^2 from day 1 to day 5 repeated every 4 weeks (arms A/B/C/D), valproic acid (VPA) p.o. from day 6 of first cycle continuously throughout all cycles (arms B/D), all-*trans* retinoic acid (ATRA) p.o. from day 6 to day 28 of each treatment cycle (arms C/D) [1]. Patients with complete remission (CR), CR without recovery of platelets or neutrophils (CRi), partial remission (PR), anti-leukemic effect (ALE) or stable disease (SD) after cycle 6 received maintenance treatment with optionally shortened DEC application from day 1 to day 3, repeated every 4 weeks.

The DECIDER study was approved by the central ethics committee (University of Freiburg). The patient's written informed consent to participate in this clinical trial was obtained before any study-specific procedures occurred.

Patients selected for the present study were required to fulfil the following criteria: (1) Achievement of CR, CRi, PR, ALE or SD on treatment. (2) Progressive disease (PD) or relapse after at least 6 months of continuous treatment, and (3) bone marrow (BM) or peripheral blood (PB) samples available both at the start of treatment and at the time of PD, referred to as resistance.

Sample preparation

Mononuclear cells were obtained from patient PB or BM samples by density gradient centrifugation using lymphocyte separation medium (PAN biotech, Aidenbach, Germany). Blasts were enriched by automated cell separation using anti-CD34 and anti-CD117 microbeads, and T cells using anti-CD3 microbeads (Miltenyi Biotec, Bergisch Gladbach, Germany). Genomic DNA was isolated with the DNeasy Blood & Tissue or QIAamp micro DNA kit (Qiagen, Hilden, Germany).

Whole exome sequencing

Blasts collected at baseline and at time of resistance were analyzed via whole exome sequencing (WES). For 11 of the 14 patients, T cell DNA was available and additionally analyzed by WES as a germline control (Supplementary Figure S1). WES data of one patient has been previously published [2].

WES was performed at the DKFZ (Heidelberg, Germany), using the SureSelect Human All Exon v7 Kit (Agilent) on a NovaSeq 6000 (Illumina, San Diego, CA, USA; paired-end 50bp). After quality control with FastQC [3] and trimming with Trimmomatic [4], variant calling was performed with Mutect2 by GATK [5] followed by false positive

filtering. Only rare (population frequency <0.1% in genomAD database), non-silent single nucleotide variants and small insertions and deletions with a variant allele frequency (VAF) greater than 5% were considered [6]. Mutations were classified with InterVar [7], ClinVar [8], cancer hotspots [9] and functional impact was predicted with the REVEL score [10]. Waterfall plots were created with the R package GenVisR [11]. The R package YAPSA [12] has been used to analyze the COSMIC mutational signatures [13]. BRCAness is the proportion of the AC03 signature. Copy number variations were determined by Control-FREEC [14]. WES data was analyzed focusing on genes known to be cancer-associated (i.e. cancer genes as defined by OncoKB) [15].

DNA methylation analysis

DNA methylation profiles were assessed using Infinium HumanMethylation450 BeadChip (n=17), Infinium MethylationEPIC BeadChip (n=10), and Infinium MethylationEPIC BeadChip v2 (n=1) arrays according to the manufacturer's (Illumina) instructions at the DKFZ (Heidelberg, Germany). All methylation array samples were processed and normalized using the *SeSAMe* [16] R package, with preprocessing performed using the "QCDPB" argument, as recommended by the *SeSAMe* documentation. Beta values were mapped to the EPIC v1 array, and probes that could not be lifted over, along with those listed in the KYCG.EPIC.Mask.20220123 database from *SeSAMe*, were excluded from the analysis. In total, the dataset contained 329 925 CpGs. To account for batch effects across different array types, batch correction was applied to the M-value matrix using the removeBatchEffect function from the *limma* R package [17].

A single-sample analysis was performed following the approach described in Greve et al. [18]. For each patient, demethylated CpGs were identified based on an absolute delta beta >0.1. The significance of overlap across 14 patients was assessed using bootstrap resampling (1 000 iterations) and ANOVA, comparing observed overlaps to randomly generated CpG lists matched for size and genomic distribution. To evaluate the overlap patterns, cubic regression models were applied: a Full model capturing distinct trajectories for measured and randomized CpGs and a Reduced model assuming a shared trajectory for both datasets. An ANOVA test was used to compare the models, with a significant p-value indicating a non-random overlap of demethylated CpGs.

Differential methylation analysis was performed using mixed linear models from SeSAMe. Multiple testing correction was applied using a smoothing-based FDR approach with empirical null modeling (fdrtool R package). CpG sites were deemed significant if they had an adjusted p-value below 0.1 and an absolute delta Beta greater than 0.1.

Cell culture

The cell lines U937, MOLM13, and OCI-AML3 were provided by the research group of Michael Lübbert (University of Freiburg) who obtained it from DSMZ (Nos. ACC 5, ACC 554 and ACC 582). Cell lines were cultured in RPMI medium plus 10% fetal bovine serum and 1% penicillin/streptomycin in a humidified 5% CO_2 atmosphere.

In order to generate cell lines with secondary DEC resistance U937 and MOLM13 were treated with increasing DEC concentrations over a period of at least six months with

regular monitoring for development of resistance. Secondary resistance was defined as a doubling of the IC_{50} compared to treatment-naïve cells in viability assays using the CellTiter-Glo® 2.0 Cell Viability Assay (Promega, Fitchburg, WI, USA). The starting concentration of DEC the cells were treated with was 100 nM. After step-wise dose escalation for about 8 months the final concentration was 80 µM. After development of secondary resistance cells were not treated for a period of two months ("drug holiday") re-inducing sensitivity against DEC treatment ("re-sensitized").

For flow cytometry analysis we cultivated the cells at 0.3x 10^6 cells per ml in 12 well plates and treated the cells with single or combination treatments of DEC (100nM), ATRA (1µM), VEN (100nM) and talazoparib (TAL) (50nM), dissolved in DMSO. The cells were treated with the respective drugs according to the treatment plan depicted in Supplementary Table S4.

Flow Cytometry

Degree of differentiation and apoptosis rate under treatment of the cell lines were assessed by flow cytometry analyses of CD11b (Biolegend, San Diego, CA, USA), CD14 (Biolegend), CD38 (Biolegend), annexin V (Miltenyi Biotec) and DAPI (Miltenyi Biotec). For this, 1x10^6 cells were harvested and centrifuged at 300g for 5 minutes. The supernatant was discarded, and the cell pellet was resuspended in 20 µL of phosphate-buffered saline (PBS) containing an Fc receptor block (prepared by diluting 2 mL Fc block in 10 mL PBS). Cells were incubated on ice for 20 minutes to minimize non-specific antibody binding. Following the blocking step, 30 µL of staining buffer containing the appropriate antibody diluted 1:50 was added to the cell suspension. The cells were incubated on ice for an additional 30 minutes. After incubation, the cells

were washed twice with PBS to remove unbound antibodies. For viability staining, DAPI was added to the final cell suspension at a 1:5 dilution. The samples were then analyzed using a BD Fortessa flow cytometer (Franklin Lakes, NJ, USA).

Supplementary data

Supplementary Table S1: Pretreatment and treatment characteristics per patient

**Patients**	**1**	**2**	**3**	**4**	**5**	**6**	**7**	**8**	**9**	**10**	**11**	**12**	**13**	**14**
**Age (years)**	76	80	75	71	72	79	71	64	76	82	73	80	73	71
**Sex**	female	male	female	male	male	male	male	male	female	male	male	male	male	male
**ECOG**	0	1	1	1	1	1	0	1	1	1	0	1	1	2
**Comorbidity Index**	2	0	0	2	0	3	0	7	5	1	8	0	3	2
**Prior hematological disorder**	no	yes	yes	no	no	no	yes	yes	yes	no	yes	no	no	yes
**Treatment related AML**	no	no	no	no	no	yes	no	no	no	no	no	no	yes	no
**White blood cell count x10⁹/L**	36.1	33.2	1.3	0.8	1.1	1.1	4.6	20.6	1.9	6.8	18.1	11.3	50.1	4.1
**Platelet count x10⁹/L**	102	124	85	34	38	121	62	25	72	48	22	36	100	492
**Serum lactate dehydrogenase U/L**	303	198	196	188	210	174	244	133	178	268	334	480	411	518
**Bone marrow blasts %**	51	30	56	42	95	25	25	90	51	46	80	20	82	50
**2022 ELN genetic risk classification**	adverse	adverse	unknown	adverse	adverse	intermediate	adverse	intermediate	intermediate	adverse	adverse	favorable	unknown	adverse
**Karyotype**	47,XX,+13[5] / 46,XX[16]	46,XY[5]	no metaphases	45,XY,+Y,del(5)(q11q31),-18,-20,-21,-22,+2mar[23]	45,XY,-7[6] 47,idem,+8,+13[2] 46,XY[12]	45,X,-Y[3] / 46,XY[17]	46,XY[20]	47,XY,+mar[14]/48,idem,+mar[5]/46,XY[1]	46,XX[20]	46,XY[20]	46,XY[20]	46,XY,t(8:21)(q22;q22)[16] / 45,idem,-Y[4]	no metaphases	44,XY,-2,t(2;6)(p23;q15),-7,del(8)(p11),der(10)t(10;17)(q26;q11),add(13)(p11),-16,-17,add(19)(q13),-22,+r,+mar[4] 88,XXYY,idemx2[3] 46,XY[15]
**Treatment**	DEC	DEC + ATRA	DEC + ATRA	DEC	DEC + ATRA	DEC + ATRA	DEC + VPA	DEC + VPA + ATRA	DEC + VPA	DEC + ATRA	DEC + ATRA	DEC + VPA	DEC + ATRA	DEC
**Time on treatment (days)**	297	906	1942	303	426	291	476	246	346	562	244	357	244	233

Supplementary Table S2: search terms in cBioPortal (accessed 08-MAY-25) [19]

Studies: Curated set of non-redundant studies →	Genomic Profiles: Mutations →	Gene: TAS2R19	Result: Mutations in 102 of 101,480 patients (including one patient with a p.G77S mutation)
Studies: ACUTE MYELOID LEUKEMIA →	Genomic Profiles: Mutations →	Gene: TAS2R19	Result: Mutations in 1 of 5 296 AML patients

Supplementary Table S3: Patient and cell line BRCAness and HRD scores at resistance

Specimen	BRCAness %	HRD
Patient 1	<1.0	7
Patient 2	27.54	8
Patient 3	85.83	9
Patient 4	17.47	9
Patient 5	73.14	7
Patient 6	83.32	14
Patient 7	85.36	6
Patient 8	70.13	2
Patient 9	<1.0	36
Patient 10	<1.0	29
Patient 11	<1.0	30
Patient 12	91.22	6
Patient 13	79.28	14
Patient 14	<1.0	98
MOLM13 Resistant	85.75	7
U937 Resensitized	82.49	42
U937 Resistant	84.05	52

Supplementary Table S4: Cell line treatment for flow cytometry analyses

Time	Day1	Day2	Day3	Day4	Day5	Day6	Day7
Treatment	DEC 100nM	DEC 100nM	DEC 100nM	VEN 100nM	Incubation	Incubation	Measure
		TAL 50nM	TAL 50nM				
			ATRA 1µM				

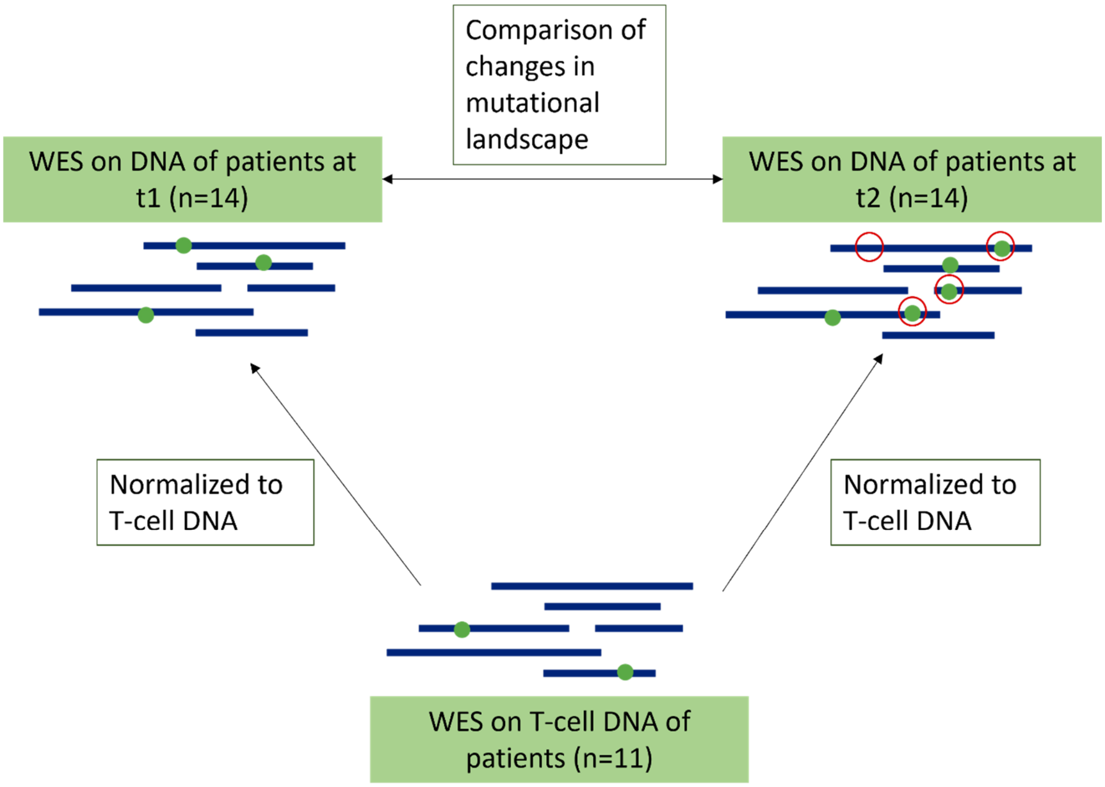

Supplementary Figure S1: Illustration of the WES data analyses applied to baseline, resistance and T cell samples.

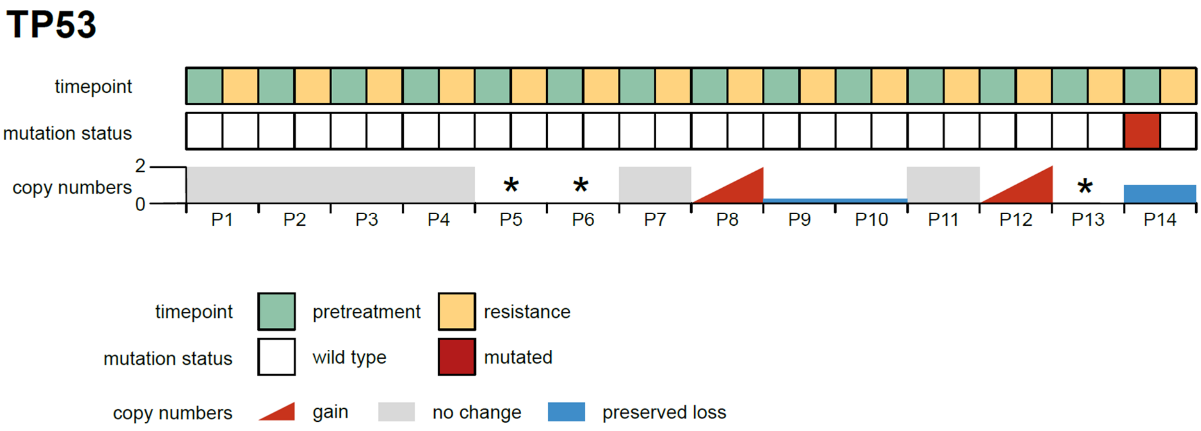

Supplementary Figure S2: Illustration of *TP53* alterations per patient at pretreatment and resistance. Timepoint, *TP53* gene mutation status and *TP53* copy numbers are indicated per patient (P). For patients without T cell reference copy numbers were not assessed (*).

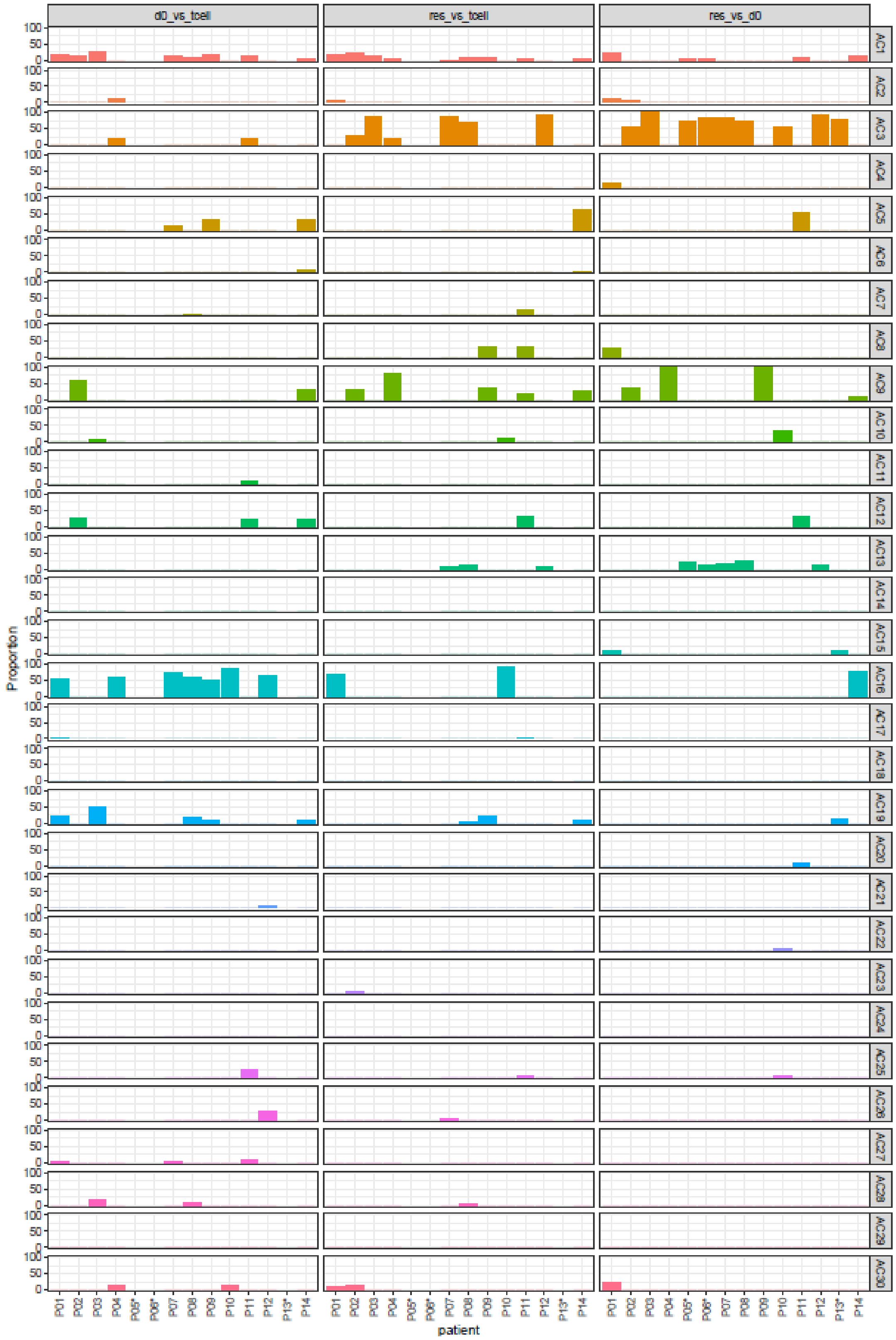

Supplementary Figure S3: Mutational signatures in patients at baseline and resistance. Patients without a T cell control are marked with an asterisk (*). Comparisons are as indicated: left panel, baseline (t1) vs. T cells; middle panel, resistance (t2) vs. T cells; right panel, t2 vs. t1. Mutational signatures were categorized according to Alexandrov et al. [13].

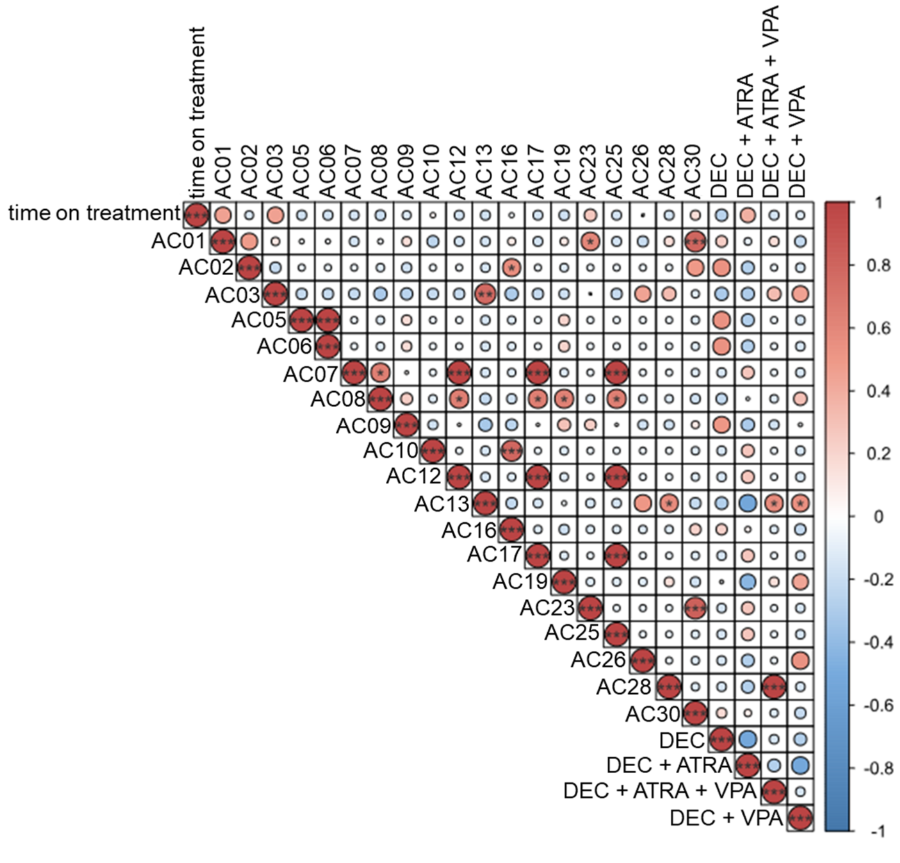

Supplementary Figure S4: Correlation analysis illustrating the relationship between treatment type, time on treatment, and the emergence of mutational signatures. The analysis identified statistically not significant positive correlation between time on treatment and additional VPA treatment with the AC03 signature. Correlation values are represented using a color gradient: red indicates positive correlations, and blue indicates negative correlations. Statistical significance is marked with asterisks: *p < 0.05, **p < 0.01, and ***p < 0.001.

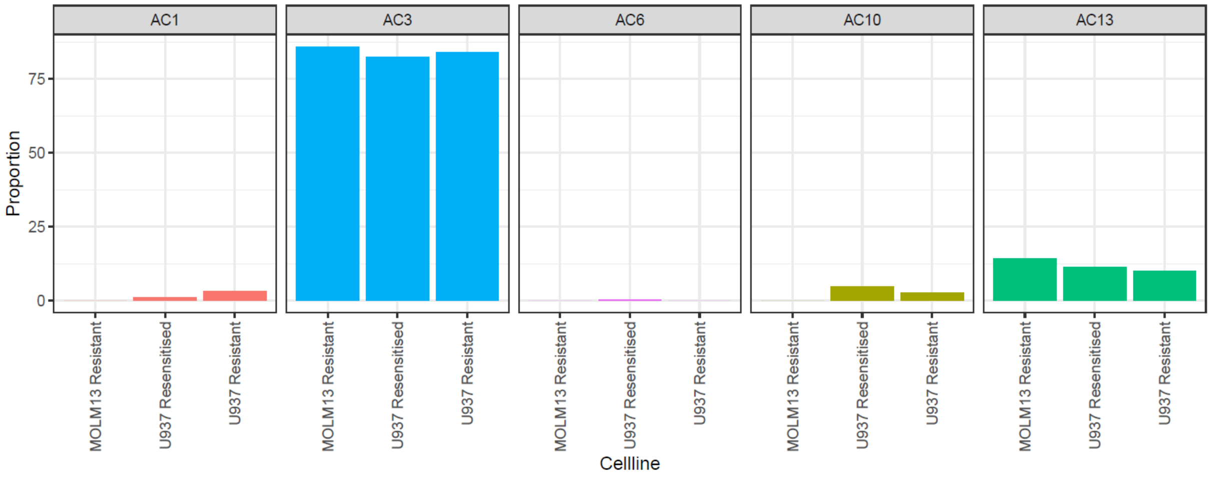

Supplementary Figure S5: Mutational signatures in cell lines as indicated compared to parental cells. Mutational signatures were categorized according to Alexandrov et al. [13].

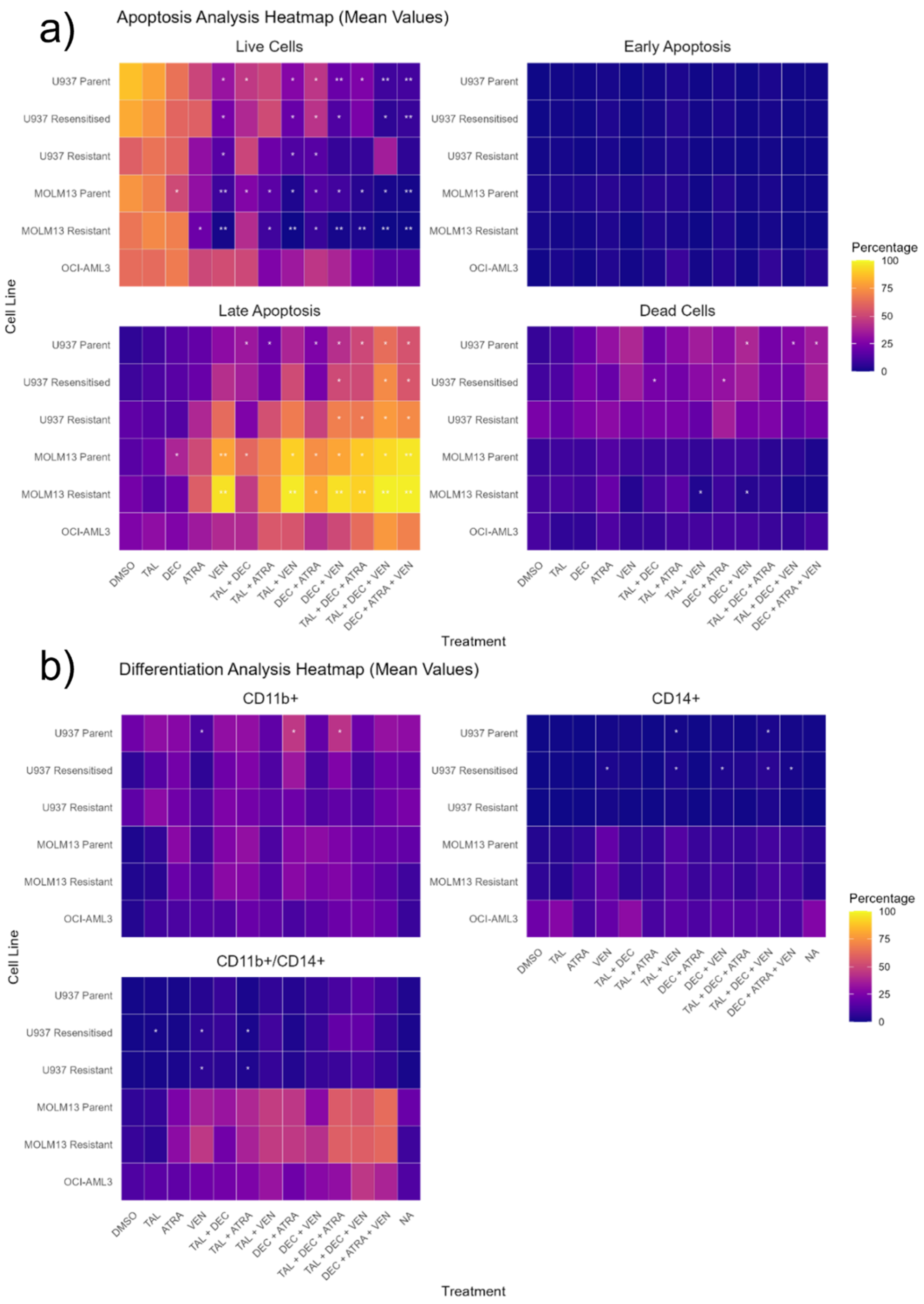

Supplementary Figure S6: Apoptosis and differentiation under treatment of parental and resistant AML cell lines. Heatmap depicting the percentage of a) cell apoptosis (live cells, early apoptosis, late apoptosis, and dead cells) and b) differentiation conditions (CD11b+, CD14+, and CD11b+/CD14+) across cell lines under treatment as indicated. Each cell condition is represented as an individual box, where brighter regions indicate higher proportions of the respective condition. All experiments were performed in triplicate (n=3). Statistical analyses were carried out using GraphPad Prism 10. A two-way ANOVA with multiple comparisons was conducted, followed by a Dunnett's test. Asterisks indicate significant changes under treatment as compared to DMSO (*<0.05; **<0.001). The data were generated using flow cytometry, with Annexin V to detect apoptotic cells and DAPI as a counterstain to identify dead cells, and CD11b and CD14, respectively.

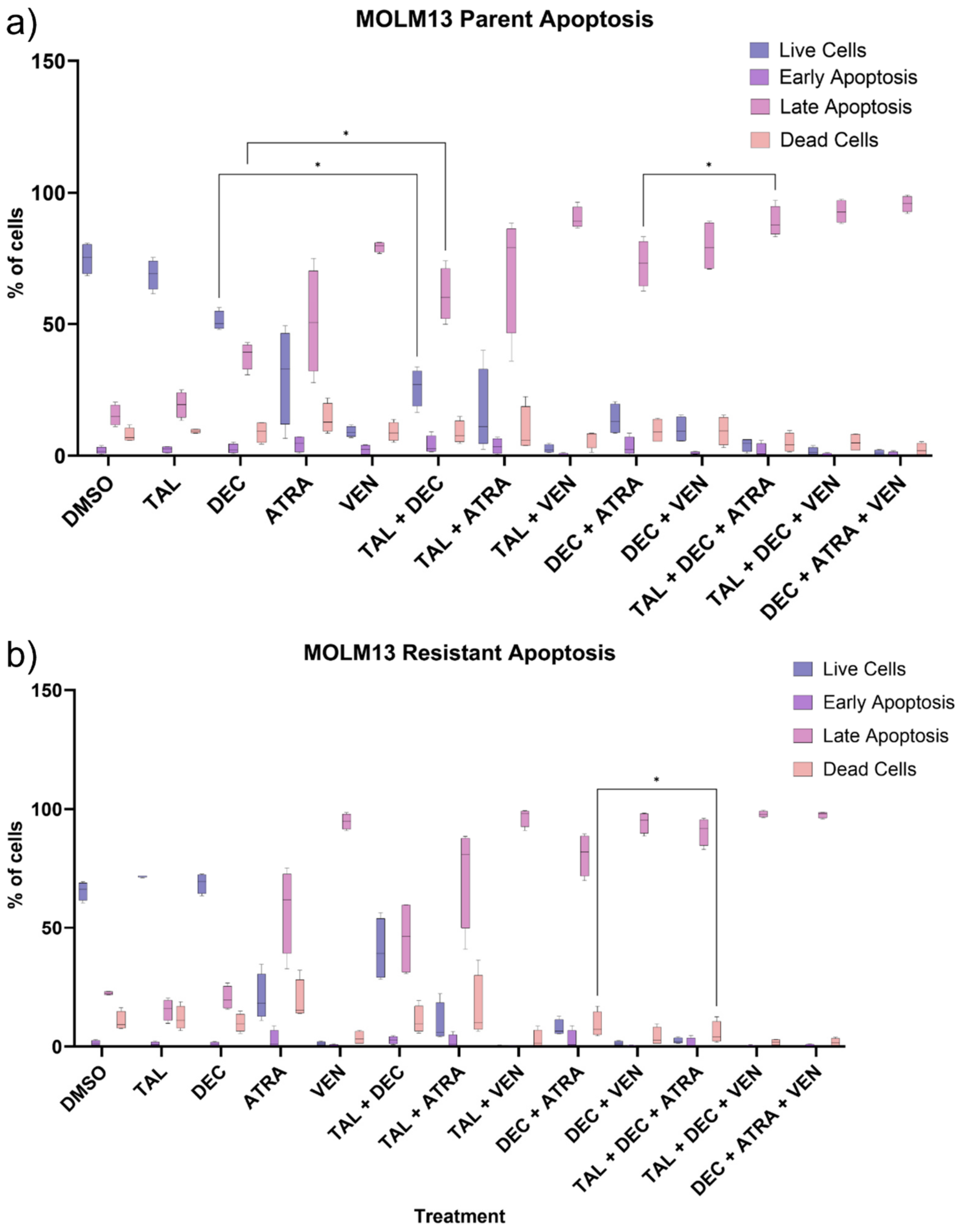

Supplementary Figure S7: Boxplots illustrating the levels of late apoptosis in a) MOLM13 DEC naïve (parental) and b) MOLM13 DEC resistant cells under the treatment as indicated. All experiments were performed in triplicate (n=3). Statistical analyses were carried out using GraphPad Prism 10. A two-way ANOVA with multiple comparisons was conducted, followed by a Tukey's test. Statistically significant differences ($P<0.05$) between treatments are indicated by asterisks (*). The y-axis represents the relative apoptotic response as percentage of cells in each indicated condition, measured across replicates, with whiskers extending to display variability within the data.

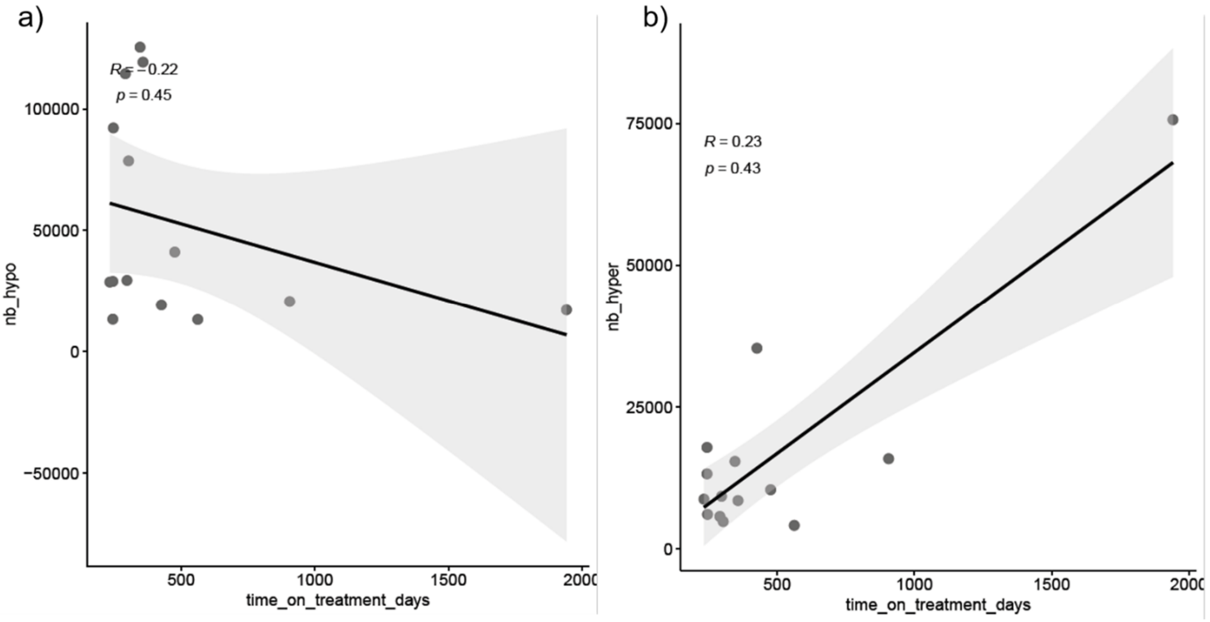

Supplementary Figure S8: Scatterplot of correlations between the number of hypo- and hypermethylated CpGs and time on treatment. a) Correlation scatterplot of hypomethylated CpGs across patients versus time on treatment. b) Scatterplot of correlation between hypermethylated CpGs and time on treatment of patients.

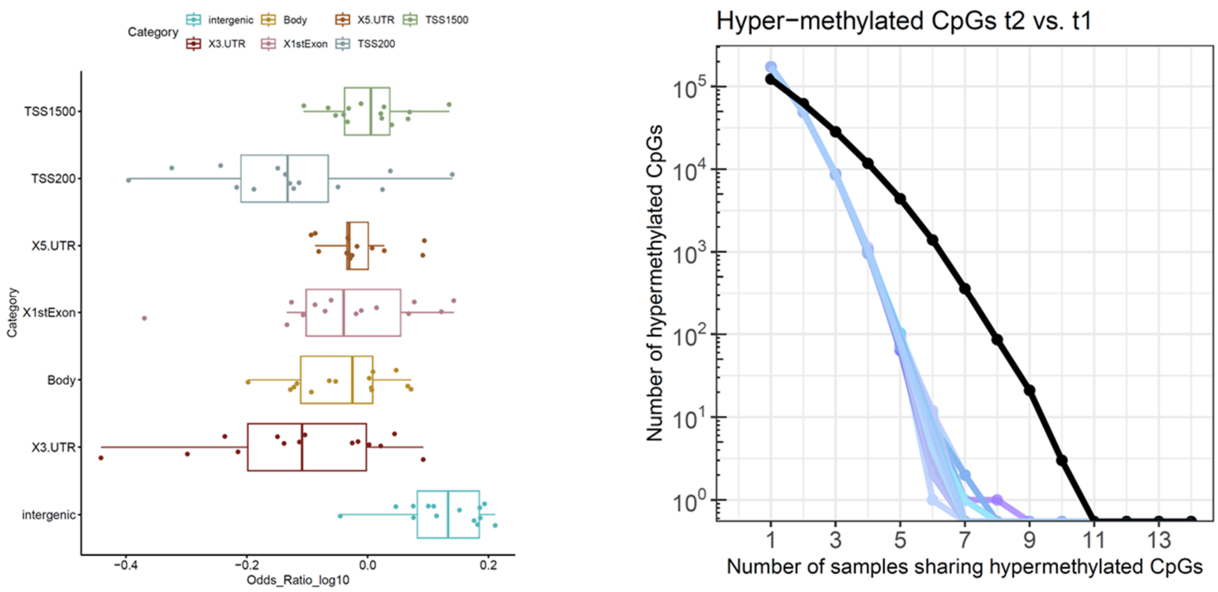

Supplementary Figure S9. (A) Genomic distribution of differentially methylated CpGs Hypermethylated sites were annotated based on their genomic context and assessed for their enrichment relative to the overall distribution of these regions in the EPIC array. Enrichment of hypermethylated CpGs of the intergenic region. (B) Identification of shared hypermethylated CpGs across patients (p value 0.52). Blue lines are random CpGs from the EPIC array, whereas the black line are CpGs commonly hypermethylated across patients.

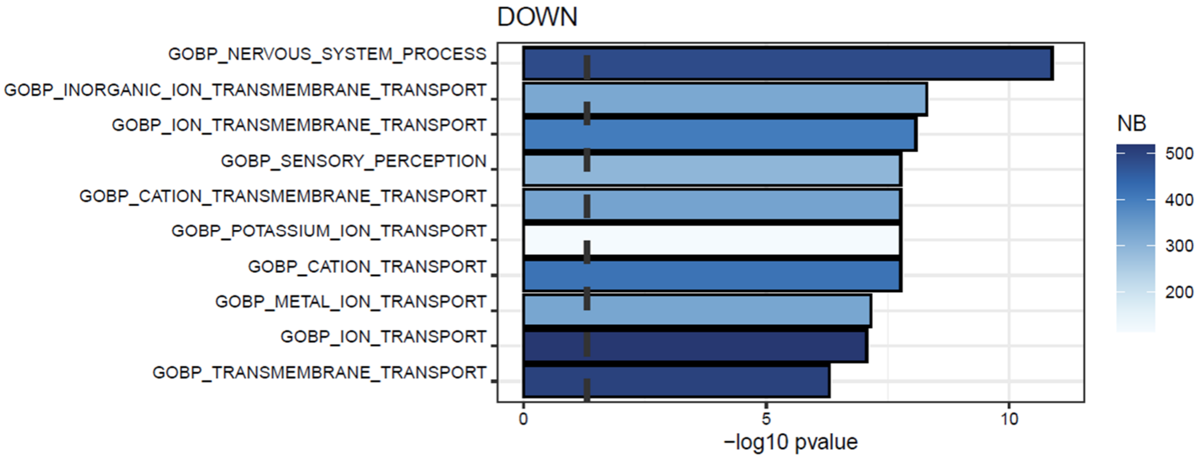

Supplementary Figure S10: Gene set enrichment analysis of DNA methylation comparing treatment start (t1) and time of resistance (t2) for GO-BP pathways. Blue bars represent hypomethylated pathways. Globally, only hypomethylated pathways were observed at t2 vs. t1. In the GO-BP pathways, significant hypomethylation was found in processes related to ion and cation transport, the nervous system, sensory perception and transmembrane transport.

References for Supplementary Data

[1] Grishina O, Schmoor C, Döhner K, Hackanson B, Lubrich B, May AM, et al. DECIDER: prospective randomized multicenter phase II trial of low-dose decitabine (DAC) administered alone or in combination with the histone deacetylase inhibitor valproic acid (VPA) and all-trans retinoic acid (ATRA) in patients >60 years with acute myeloid leukemia who are ineligible for induction chemotherapy. BMC Cancer 2015; 15: 430.

[2] Javorniczky NR, Grishina O, Hund I, Pantic M, Pfeifer D, Schmoor C, et al. Long-term decitabine/retinoic acid maintenance treatment in an elderly sAML patient with high-risk genetics. Clin Epigenetics. 2023;15:185.

[3] Babraham Bioinformatics - FastQC A Quality Control tool for High Throughput Sequence Data, https://www.bioinformatics.babraham.ac.uk/projects/fastqc/ (accessed 4 June 2025).

[4] Bolger AM, Lohse M, Usadel B. Trimmomatic: a flexible trimmer for Illumina sequence data. Bioinforma Oxf Engl 2014; 30: 2114–2120.

[5] Van der Auwera GA, O´Connor BD. Genomics in the cloud : using Docker, GATK, and WDL in Terra. First Edition. Sebastopol, CA: O´Reilly Media, 2020.

[6] Karczewski KJ, Francioli LC, Tiao G, Cimmungs BB, Alföldi J, Wang Q, et al. The mutational constraint spectrum quantified from variation in 141,456 humans. Nature 2020; 581: 434–443.

[7] Li Q, Wang K. InterVar: Clinical Interpretation of Genetic Variants by the 2015 ACMG-AMP Guidelines. Am J Hum Genet 2017; 100: 267–280.

[8] Landrum MJ, Lee JM, Riley GR, Jang W, Rubinstein WS, Church DM, et al. ClinVar: public archive of relationships among sequence variation and human phenotype. Nucleic Acids Res 2014; 42: D980–D985.

[9] Chang MT, Bhattarai TS, Schram AM, Bielski CM, Donoghe MTA, Jonsson P, et al. Accelerating Discovery of Functional Mutant Alleles in Cancer. Cancer Discov 2018; 8: 174–183.

[10] Ioannidis NM, Rothstein JH, Pejaver V, Middha S, McDonnell SK, Baheti S, et al. REVEL: An Ensemble Method for Predicting the Pathogenicity of Rare Missense Variants. Am J Hum Genet 2016; 99: 877–885.

[11] Skidmore ZL, Wagner AH, Lesurf R, Campbell KM, Kunisaki J, Griffith OL, et al. GenVisR: Genomic Visualizations in R. Bioinforma Oxf Engl 2016; 32: 3012–3014.

[12] Hübschmann D, Jopp-Saile L, Andresen C, Krämer s, Gu Z, Heilig CE, et al. Analysis of mutational signatures with yet another package for signature analysis. Genes Chromosomes Cancer 2021; 60: 314–331.

[13] Alexandrov LB, Nik-Zainal S, Wedge DC, Aparicio SA, Behjati S, Biankin AV, et al. Signatures of mutational processes in human cancer. Nature 2013; 500: 415–421.

[14] Boeva V, Popova T, Bleakley K, Chiche P, Cappo J, Schleiermacher G, et al. Control-FREEC: a tool for assessing copy number and allelic content using next-generation sequencing data. Bioinforma Oxf Engl 2012; 28: 423–425.

[15] Chakravarty D, Gao J, Phillips S, Kundra R, Zhang H, Wang J, et al. OncoKB: A Precision Oncology Knowledge Base. JCO Precis Oncol 2017; 1–16.

[16] Zhou W, Triche TJ Jr, Laird PW, Shen H. SeSAMe: reducing artifactual detection of DNA methylation by Infinium BeadChips in genomic deletions. Nucleic Acids Res 2018; 46: e123.

[17] Ritchie ME, Phipson B, Wu D, Hu Y, Law CW, Shi W, et al. limma powers differential expression analyses for RNA-sequencing and microarray studies. Nucleic Acids Res 2015; 43: e47.

[18] Greve G, Andrieux G, Schlosser P, Blagitko-Dorfs N, Rehman U, Ma T, et al. In vivo kinetics of early, non-random methylome and transcriptome changes induced by DNA-hypomethylating treatment in primary AML blasts. Leukemia 2023; 37: 1018–1027.

[19] Cerami E, Gao J, Dogrusoz U, Gross B, Sumer SO, Aksoy BA, et al. The cBio cancer genomics portal: an open platform for exploring multidimensional cancer genomics data. Cancer Discov. 2012;2:401–404.
